# Real-world data of sex differences in atrial fibrillation catheter ablation: Insights from a prospective multicenter registry

**DOI:** 10.1016/j.hroo.2026.04.005

**Published:** 2026-04-13

**Authors:** Zhuxin Zhang, Daniela Hincapie, Christopher Thorne, Allyson Varley, Linda Justice, Jose Osorio, Anthony Moretta, Joshua Silverstein, Anil Rajendra, Gustavo Morales, Sandeep Goyal, Yaariv Khaykin, Mouhannad Sadek, Benjamin D’Souza, Imdad Ahmed, Mohammad Ali Jazayeri, Subha Varahan, Matthew Sackett, Julia McHugh, Alexandru Costea, Ann Lowery Black, Yan Yao, William H. Sauer, Paul C. Zei, REAL-AF Investigators

**Affiliations:** 1 Cardiac Arrhythmia Service, Brigham and Women’s Hospital and Harvard Medical School, Boston, Massachusetts; 2 Arrhythmia Center, State Key Laboratory of Cardiovascular Disease, Fuwai Hospital, National Center for Cardiovascular Diseases, Chinese Academy of Medical Sciences and Peking Union Medical College, Beijing, China; 3 Department of Cardiology, Zhongshan Hospital, Fudan University, Shanghai Institute of Cardiovascular Diseases, Shanghai, China; 4 Heart Rhythm Clinical and Research Solutions, Birmingham, Alabama; 5 Cardiac Arrhythmia Service, HCA Florida, Miami, Florida; 6 Cardiac Arrhythmia Service, Sarasota Memorial Hospital, Sarasota, Florida; 7 Cardiac Arrhythmia Service, Allegheny Health Network, Pittsburgh, Pennsylvania; 8 Cardiac Arrhythmia Service, Arrhythmia Institute at Grandview, Birmingham, Alabama; 9 Cardiac Arrhythmia Service, Piedmont Hospital, Atlanta, Georgia; 10 Cardiac Arrhythmia Service, Southlake Regional Health Centre, Ontario, Canada; 11 Perelman School of Medicine at the University of Pennsylvania, Philadelphia, Pennsylvania; 12 Cardiac Arrhythmia Service, MercyHealth, Janesville, Wisconsin; 13 Cardiac Arrhythmia Service, Trinity Health Michigan Heart-Ann Arbor Campus, Ypsilanti, Michigan; 14 Cardiac Arrhythmia Service, Oklahoma Heart Hospital, Oklahoma City, Oklahoma; 15 Cardiac Arrhythmia Service, Centra Heart and Vascular Institute, Lynchburg, Virginia; 16 Cardiac Arrhythmia Service, The Christ Hospital Health Network, Cincinnati, Ohio

**Keywords:** Atrial fibrillation, Catheter ablation, Quality improvement, Ablation strategy, Women’s health

## Abstract

**Background:**

Sex differences in atrial fibrillation (AF) ablation strategies and outcomes remain insufficiently characterized.

**Objective:**

This study aimed to evaluate the impact of sex on ablation strategies and clinical outcomes in patients with paroxysmal (PAF) and persistent AF (PsAF) in real-world settings.

**Methods:**

Data from the REAL-AF registry were analyzed (January 2018 to May 2023). Demographics, intraoperative parameters, and outcomes were compared by sex, stratified by AF type.

**Results:**

A total of 4202 patients (41.1% women) were included. Women were older (69.02 ± 9.18 vs 65.32 ± 10.49 years), had higher CHA_2_DS_2_-VASc scores (3.36 ± 1.43 vs 2.33 ± 1.45), and were more frequently diagnosed as having PAF (72.8% vs 61.3%) (*P* < .001 for all). Across both AF types, women underwent more complex ablation procedures, including additional substrate modification (SM) (pulmonary vein isolation [PVI] + cavotricuspid isthmus [CTI] + SM: 25.8% vs 22.7%; *P* = .020), particularly posterior wall isolation (53.9% vs 45.7% in PsAF; 18.2% vs 12.3% in PAF). Generalized linear mixed models revealed similar acute success (first-pass PVI) between sexes; however, women required more repeat ablations after the blanking period (7.2% vs 5.5%; *P* = .022). Multivariable Cox analysis showed that women had a significantly higher long-term recurrence risk (adjusted hazard ratio 1.24; 95% confidence interval 1.06–1.45; *P* = .009). Subgroup analysis identified female sex as an independent predictor of recurrence in ablation strategies incorporating SM, whereas PVI-only and PVI + CTI approaches yielded comparable outcomes. Complication rates were comparable, but women with PsAF had a higher incidence of vascular access complications (1.3% vs 0.1%; *P* = .010).

**Conclusion:**

Women face higher long-term arrhythmia recurrence risks, especially in ablation strategies involving SM, despite comparable acute and short-term outcomes.

**Clinical trial registration:**

NCT04088071

## Graphical abstract

**Figure:**
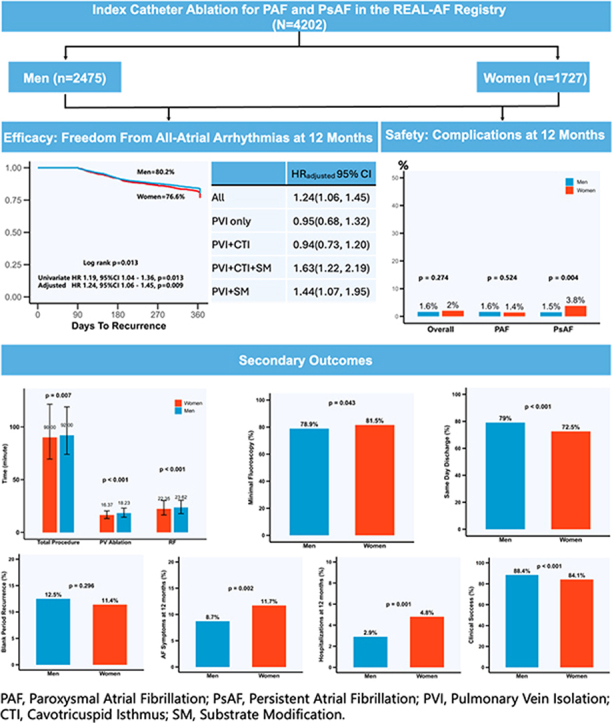

**Key Findings**

▪ In a real-world cohort of 4202 patients undergoing atrial fibrillation (AF) ablation, women were older, had higher CHA_2_DS_2_-VASc scores, and were more frequently diagnosed as having paroxysmal AF than men.
▪ Women underwent more complex ablation procedures, including higher rates of additional substrate modification—particularly posterior wall isolation—across both paroxysmal and persistent AF.
▪ Despite similar acute procedural success, women experienced a higher risk of long-term atrial arrhythmia recurrence and were more likely to undergo repeat ablation after the blanking period.
▪ Female sex was an independent predictor of recurrence in ablation strategies incorporating substrate modification, whereas outcomes were comparable between sexes with pulmonary vein isolation–based approaches alone.

## Introduction

Catheter ablation (CA) has become the primary rhythm control strategy for atrial fibrillation (AF), yet studies consistently report differences in ablation outcomes between men and women.^1^^,^^2^ Women with AF are often older at the time of ablation, present with more severe symptoms, and have more advanced atrial remodeling than men,^3^^,^^4^ which might affect procedural success and increase complication rates.^5^, ^6^, ^7^ Recognizing these physiological differences, recent guidelines emphasize the importance of individualized ablation strategies based on patient characteristics, including sex.^6^, ^7^, ^8^

Despite these recommendations, understanding these sex-specific differences is challenging owing to the underrepresentation of women in randomized controlled trials. Women constitute only 12%–26% of participants in most AF trials,^9^, ^10^, ^11^ which limits the generalizability of the findings and may underestimate the unique challenges they face. Moreover, randomized controlled trials often exclude patients with significant comorbidities or advanced disease—conditions more prevalent among women.^12^ In contrast, previous large-scale studies have predominantly relied on health care claims data or electronic health records, lacking detailed clinical information on patient characteristics and procedural nuances.^13^, ^14^, ^15^, ^16^

Therefore, real-world data (RWD) from large-scale registries, such as the REAL-AF registry, offer invaluable insights that complement controlled clinical trials by capturing the heterogeneity of patient populations and clinical practices across diverse settings. The REAL-AF registry, a multicenter prospective study, has provided detailed evaluations of ablation outcomes in both patients with paroxysmal (PAF) and persistent AF (PsAF),^17^ with sex differences being a central theme that requires more comprehensive exploration. This study sought to fill that gap by providing an in-depth analysis of sex-specific differences in baseline characteristics, ablation strategies, perioperative management, complications, and clinical outcomes in patients with AF. Although previous research has typically focused on 1 or 2 aspects, often from distinct cohorts or populations,^18^, ^19^, ^20^ the current study leverages large-scale RWD to provide a more holistic view.

## Methods

### Study design and population

This analysis is derived from the REAL-AF registry (NCT04088071), a large, prospective, multicenter study designed to collect RWD on outcomes of radiofrequency CA (RFCA) in patients with PAF and PsAF. The registry includes patients who underwent their first RFCA between January 2021 and July 2023 at 44 centers across the United States, involving 114 operators. This broad representation of clinical practice allows flexibility in clinical protocols, whereas standardized data collection procedures ensure consistency across centers. Eligible participants were adults aged ≥18 years, diagnosed as having PAF or PsAF, confirmed by electrocardiogram or ambulatory monitoring, and scheduled for their first RFCA procedure. Patients with previous ablation procedures or contraindications to RFCA were excluded. Baseline demographic and clinical data were collected using standardized case report forms and entered into an electronic data management system.

### Ablation procedure

All individuals included in the study underwent RFCA for PAF or PsAF under general anesthesia (GA), monitored anesthesia care, or moderate sedation, with either uninterrupted oral anticoagulation (OAC) or minimally interrupted OAC (direct OAC held on the morning of the procedure). Operators had the autonomy to decide whether to use ultrasound-guided venous access. Ablation procedures were performed using a 3-dimensional electroanatomic mapping system (CARTO® 3, Biosense Webster, Irvine, CA) to create detailed maps of the left atrium (LA), pulmonary veins (PVs), and posterior wall (PW). Areas of low voltage and the extent of scarring were recorded based on the operator’s visual estimate. The ablation strategy primarily involved wide-area circumferential PV isolation (PVI). Energy settings varied based on operator preference, using 1 of 2 approaches: (1) standard-power, long-duration (25–35 W, 30–60 seconds per lesion) or (2) high-power, short-duration (40–50 W, <20 seconds per lesion) ablation. Catheter stability and lesion formation were monitored in real time, maintaining contact force between 10 and 20 g. Additional substrate modification (SM), such as PW isolation (PWI) or non-PV trigger ablation, was performed as needed. Procedures were done under zero fluoroscopy, minimal fluoroscopy, defined as <2 minutes of fluoroscopy, or conventional fluoroscopy, defined as ≥2 minutes of fluoroscopy.

### Follow-up and outcome measures

Patients were followed at 3 and 12 months after ablation, with additional follow-up as needed for symptomatic arrhythmia recurrence. Reasons for OAC discontinuation were recorded as open-ended text and were not the focus of the current analysis. However, representative responses are presented in Supplemental Table 1. Routine evaluations included 12-lead electrocardiograms at each visit, alongside continuous rhythm monitoring using ambulatory monitors, consumer wearable devices, or implantable loop recorders at 6 and 12 months. The primary outcome was freedom from atrial arrhythmia (AF, atrial flutter [AFL], or atrial tachycardia [AT]) lasting ≥30 seconds at 12 months, after a 90-day blanking period. Recurrence was defined as any AF, AFL, or AT episode recorded after the blanking period. Secondary outcomes included procedural metrics such as total procedure time, radiofrequency (RF) application time, and success of PVI. Procedural complications, both acute and long-term, were evaluated, including pericardial effusion, phrenic nerve injury, and PV stenosis.

### Statistical analysis

Continuous variables were tested for normality with the Shapiro-Wilk test and presented as mean ± standard deviation or median [Q1–Q3], depending on the distribution. Group comparisons were made using Student *t* test for normally distributed variables and the Mann-Whitney U test for skewed data. Categorical variables were analyzed using the χ^2^ test or Fisher’s exact test, as appropriate. Generalized linear mixed models were used to assess dichotomous endpoints. Kaplan-Meier survival curves evaluated freedom from atrial arrhythmia at 12 months, with sex differences assessed using the log-rank test. Cox proportional hazards models were applied to assess sex differences in arrhythmia recurrence, adjusting for age, body mass index, AF type, and comorbidities, with hazard ratios (HRs) and 95% confidence intervals (CIs) reported. Variance inflation factor values were all below 2.0, indicating no multicollinearity issues. The proportional hazards assumption was confirmed using the Schoenfeld test. Subgroup analyses were conducted by AF type (PAF vs PsAF). Patients lost to follow-up were excluded from survival and regression analyses. *P* < .05 was considered statistically significant. Data were analyzed using IBM SPSS (version 29.0) and R Studio (2023.06.0).

### Ethical considerations

The REAL-AF registry received initial approval from the Western Copernicus Group™ Institutional Review Board on October 31, 2018. Informed consent was obtained from all participants before enrollment, unless a waiver of consent was granted by the participating center, in accordance with the principles of the Declaration of Helsinki.

## Results

### Study population

The study included 4202 patients with AF who underwent their first RFCA (Supplemental Figure 1), with 2773 (66%) diagnosed as having PAF and 1429 (44%) PsAF. Overall, 41.2% were women (45.3% in PAF; 32.9% in PsAF). Women in both AF groups were significantly older than men at the time of ablation. Interestingly, although women had higher CHA_2_DS_2_-VASc scores, they exhibited significantly lower rates of cardiovascular comorbidities. LA diameter was consistently smaller in women across both groups, alongside a higher left ventricular ejection fraction. Despite these, a higher rate of previous stroke/transient ischemic attack was seen in women. Women also had a higher frequency of hospital or emergency room visits and antiarrhythmic drug (AAD) failures, indicating more frequent health care utilization before ablation. In terms of medication use, the profiles of men and women were mostly similar across AADs and OAC. However, men were more likely to be on aspirin (Table 1).

**Table 1:** Patient baseline characteristics

Characteristics	Overall (N = 4202)			PAF (n = 2773)			PsAF (n = 1429)		
	Men (n = 2475)	Women (n = 1727)	*P* value	Men (n = 1516)	Women(n = 1257)	*P* value	Men(n = 959)	Women(n = 470)	*P* value
Age at procedure, y	65.32 ± 10.49	69.02 ± 9.18	<.001	64.16 ± 11.06	68.11 ± 9.57	<.001	67.17 ± 9.2	71.44 ± 7.53	<.001
Race									
American Indian or Alaska Native	5, 0.2	4, 0.2	.838	2, 0.1	3, 0.2	.510	3, 0.3	1, 0.2	.737
Asian	31, 1.3	25, 1.4	.587	11, 1.5	18, 1.4	.966	9, 0.9	7, 1.5	.352
Black or African American	97, 3.9	76, 4.4	.440	59, 3.9	50, 4.0	.908	38, 4.0	26, 5.5	.178
Native Hawaiian or other Pacific Islander	5, 0.2	1, 0.1	.224	4, 0.3	1, 0.1	.255	1, 0.1	0, 0.0	.484
Other/unknown	42, 1.7	32, 1.9	.705	28, 1.8	21, 1.7	.726	114, 1.5	11, 2.3	.233
Body mass index, kg/m^2^	31.15 ± 6.40	30.97 ± 7.61	.405	30.65 ± 6.19	30.40 ± 7.147	.330	31.94 ± 6.63	32.47 ± 8.55	.121
CHA_2_DS_2_-VASc score	2.33 ± 1.45	3.36 ± 1.43	<.001	2.13 ± 1.43	3.17 ± 1.39	<.001	2.66 ± 1.42	3.86 ± 1.41	<.001
Years since AF was first diagnosed	2.94 ± 4.33	2.96 ± 4.60	.874	3.02 ± 4.47	3.03 ± 4.77	.958	2.8 ± 4.09	2.77 ± 4.1	.887
Preprocedure AF treatment history									
Cardioversion	892, 36.6	507, 29.7	<.001	281, 18.8	209, 16.8	.176	611, 64.59	298, 64.1	.863
Hospital/ER visit	808, 33.3	623, 36.8	.022	489, 32.8	463, 37.6	.010	319, 34.04	160, 34.6	.828
Previously failed AAD	558, 22.6	455, 26.4	.015	334, 22.0	315, 25.1	.167	224, 23.36	140, 29.8	.022
Medical history									
Congestive heart failure	512, 20.7	250, 14.5	<.001	209, 13.8	121, 9.6	.001	303, 31.69	129, 27.4	.101
Hypertension	1841, 74.5	1192, 69.0	<.001	1062, 70.1	818, 65.1	.004	779, 81.49	374, 79.6	.389
Diabetes	509, 20.6	291, 16.9	.002	286, 18.9	174, 13.8	<.001	223, 23.34	117, 24.9	.514
TIA/CVA	200, 8.1	189, 10.9	.002	120, 7.9	135, 10.7	.011	80, 8.37	54, 11.5	.058
Vascular disease	573, 23.2	184, 10.7	<.001	346, 22.9	134, 10.7	<.001	227, 23.74	50, 10.6	<.001
Renal disease	139, 5.6	56, 3.3	<.001	75, 5.0	33, 2.6	.002	64, 6.69	23, 4.9	.188
Liver disease	22, 0.9	15, 0.9	.950	13, 0.9	5, 0.4	.135	9, 0.94	10, 2.1	.065
Sleep Apnea	1183, 49.2	597, 35.7	<.001	722, 48.9	425, 35.1	<.001	461, 49.62	172, 37.3	<.001
Left ventricular ejection fraction	53.998 ± 13.21	57.01 ± 9.63	<.001	55.82 ± 13.51	58.35 ± 8.45	<.001	51.24 ± 112.25	53.60 ± 11.48	.002
Medication history									
Amiodarone	407, 16.8	266, 15.8	.373	184, 12.4	149, 12.1	.829	223, 23.75	117, 25.4	.490
Dofetilide	34, 1.4	22, 1.3	.785	15, 1.0	14, 1.1	.746	19, 2.02	8, 1.7	.717
Dronedarone	91, 3.8	66, 3.9	.802	71, 4.8	53, 4.3	.558	20, 2.13	13, 2.8	.420
Flecainide	314, 13.0	269, 15.9	.007	234, 15.8	219, 17.8	.153	80, 8.52	50, 10.9	.155
Propafenone	54, 2.2	41, 2.4	.676	40, 2.7	36, 2.9	.713	14, 1.49	5, 1.1	.540
Sotalol	165, 6.8	119, 7.0	.768	99, 6.7	96, 7.8	.252	66, 7.03	23, 5.0	.144
Beta-blockers/CCB (class II or IV only)	1782, 72	1256, 72.7	.628	1053, 69.6	887, 70.7	.533	729, 76.16	369, 78.5	.325
Aspirin	631, 25.5	318, 18.5	<.001	41, 27.4	246, 19.6	<.001	216, 22.52	72, 15.4	.002

Values are presented as means ± standard deviations and numbers, percentages.

AAD = antiarrhythmic drug; AF = atrial fibrillation; CCB = calcium channel blocker; CVA = cerebrovascular accident; ER = emergency room; PAF = paroxysmal atrial fibrillation; PsAF = persistent atrial fibrillation; TIA = transient ischemic attack.

### Electroanatomic and procedural characteristics

**Figure 1:**
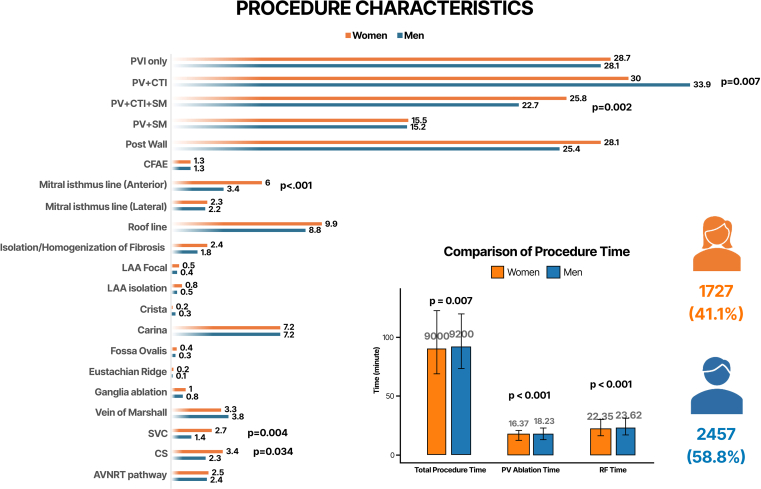
Ablation strategy and procedure time by sex during ablation for the entire cohort. CFAE = complex fractionated atrial electrograms; CS = coronary sinus; CTI = cavotricuspid isthmus ablation; LAA = left atrial appendage; PV = pulmonary vein; PVI = pulmonary vein isolation; SM = substrate modification; SVC = superior vena cava.

As shown in Table 2, presenting rhythm differed significantly by sex in the overall cohort, with women more frequently presenting in sinus rhythm than men (69.5% vs 61.3%; *P* < .001). Conversely, men more frequently presented with AF (33.4% vs 28.3%; *P* = .001) and typical AFL (6.1% vs 2.2%; *P* < .001). Atypical AFL trended to be more frequent in women (1.6% vs 1.0%; *P* = .083), reaching statistical significance in the PAF cohort (1.4% vs 0.5%; *P* = .012). Mapping results revealed that women had significantly smaller LA volume (93.5 ± 31.9 mL vs 109.9 ± 38.2 mL; *P* < .0001) and a higher LA scarring area (median 5.00% [0.00–20.00] vs 2.00% [0.00–10.00]; *P* < .001) than men (Table 2). Overall, women received more additional ablation beyond PVI. In the PsAF cohort, women were more likely to undergo PWI (53.9% vs 45.7%; *P* = .002) and linear ablation (mitral isthmus anterior 11.7% vs 6.1%, *P* < .001; roof line 21.2% vs 14.7%, *P* = .002). Similarly, in the PAF cohort, women more frequently required SM beyond PVI and additional superior vena cava and coronary sinus ablation. In contrast, men were more likely to receive cavotricuspid isthmus (CTI) ablation (Figure 1, Table 2). Ablation strategy and procedure characteristics stratified by AF type are presented in Supplemental Table 2 and Supplemental Figures 2 (PAF) and 3 (PsAF).

**Table 2:** Procedure characteristics and ablation strategy by sex and AF type

Characteristics	Overall			PAF			PsAF		
	Men	Women	*P* value	Men	Women	*P* value	Men	Women	*P* value
Thrombus detection									
TEE	946 (38.3)	710 (41.2)	.056	587 (38.7)	524 (41.8)	.108	359 (37.5)	186 (39.7)	.425
ICE	1160 (46.9)	783 (45.4)	.341	774 (48.9)	588 (46.9)	.280	419 (43.7)	195 (41.6)	.439
Cardiac CT	224 (9.1)	150 (8.7)	.690	129 (8.5)	106 (8.4)	.949	95 (9.9)	44 (9.4)	.749
Not Completed	355 (14.4)	203 (11.8)	.015∗	187 (12.3)	130 (10.4)	.102	168 (17.5)	73 (15.6)	.350
Catheter type									
Thermocool SF	5 (0.2)	6 (0.3)	.376	4 (0.3)	5 (0.4)	.740	1 (0.1)	1 (0.2)	.550
SmartTouch	15 (0.6)	13 (0.8)	.565	9 (0.6)	6 (0.5)	.678	6 (0.6)	7 (1.5)	.137
SmartTouch SF	2455 (99.2)	1708 (98.9)	.331	1503 (99.1)	1246 (99.1)	.960	952 (99.3)	462 (98.3)	.102
Ablation catheter sheath									
Short	147 (5.9)	105 (6.1)	.850	114 (7.5)	89 (7.1)	.658	33 (3.4)	16 (3.4)	.971
Long fixed	219 (8.8)	141 (8.2)	.436	141 (9.3)	106 (8.4)	.424	78 (8.1)	35 (7.4)	.651
Steerable	2058 (83.2)	1443 (83.6)	.730	1210 (79.8)	1024 (81.5)	.275	848 (88.4)	419 (89.1)	.685
Anesthesia method									
GA	2239 (90.6)	1504 (87.1)	<.001∗	1292 (85.4)	1038 (82.6)	.049∗	947 (98.7)	466 (99.1)	.499
Conscious Sedation	34 (1.5)	23 (1.5)	.998	22 (1.6)	20 (1.9)	.705	12 (1.3)	3 (0.6)	.428
Ventilation mode									
Standard	1456 (63.5)	957 (61.7)	.266	850 (63.7)	673 (62.3)	.474	606 (63.2)	284 (60.4)	.311
HFLV	910 (39.7)	690 (44.5)	.003∗	511 (38.3)	466 (43.1)	.016∗	399 (41.6)	224 (47.7)	.030∗
JET	236 (10.3)	143 (9.2)	.276	119 (8.9)	84 (7.8)	.314	117 (12.2)	59 (12.6)	.849
Intermittent apnea	53 (2.3)	20 (1.3)	.023∗	25 (1.9)	14 (1.3)	.263	28 (2.9)	6 (1.3)	.056
Presenting rhythm									
AF	814 (33.4)	481 (28.3)	.001∗	238 (16.1)	174 (14.2)	.164	576 (60.1)	307 (65.3)	.055
Typical AFL	149 (6.1)	37 (2.2)	<.001∗	76 (5.1)	20 (1.6)	<.001∗	73 (7.6)	17 (3.6)	.003∗
Atypical AFL	24 (1.0)	27 (1.6)	.083	7 (0.5)	17 (1.4)	.012∗	17 (1.8)	10 (2.1)	.643
Sinus rhythm	1494 (61.3)	1180 (69.5)	<.001∗	1170 (79.2)	1031 (84.0)	.001∗	324 (33.8)	149 (31.7)	.432
AT	5 (0.2)	9 (0.5)	.077	2 (0.1)	8 (0.7)	.059	3 (0.3)	1 (0.2)	1
SVT	4 (0.1)	2 (0.1)	1	1 (0.1)	2 (0.2)	.872	1 (0.1)	0 (0.0)	1
Other	30 (1.2)	12 (0.7)	.098	18 (1.2)	10 (0.8)	.302	12 (1.3)	2 (0.4)	.229
Mapping									
LA volume, mL	109.91 ± 38.16	93.47 ± 31.91	<.001∗	100.35 ± 33.07	87.51 ± 28.52	<.001∗	124.96 ± 40.72	109.57 ± 34.93	<.001∗
LA volume index, mL/m^2^†	49.46 ± 17.42	48.37 ± 16.68	.046∗	45.55 ± 15.27	45.65 ± 14.92	.868	55.62 ± 18.77	55.68 ± 18.84	.952
Any scarring area (≥1%)	868 (47.0)	750 (54.2)	<.001∗	400 (36.2)	458 (46.2)	<.001∗	468 (63.2)	292 (74.3)	<.001∗
LA scarring, %	2.00 (0.00–10.00)	5.00 (0.00–20.00)	<.001∗	0.00 (0.00–5.00)	3.00 (0.00–10.00)	<.001∗	5.00 (1.00–20.00)	15.00 (5.00–40.00)	<.001∗
RF strategy									
PVI only, %	696 (28.1)	495 (28.7)	.702	518 (34.2)	439 (34.9)	.677	178 (18.6)	56 (11.9)	.001∗
PV + CTI	840 (33.9)	518 (30.0)	.007∗	618 (40.8)	434 (34.5)	.001∗	222 (23.1)	84 (17.9)	.022∗
PV + CTI + SM	561 (22.7)	445 (25.8)	.020∗	241 (15.9)	242 (19.3)	.020∗	320 (33.4)	203 (43.2)	<.001∗
PV+SM	376 (15.2)	267 (15.5)	.812	137 (9.0)	140 (11.1)	.066	239 (24.9)	127 (27.0)	.393
Additional lesion sets									
Posterior wall	606 (25.4)	467 (28.1)	.051	178 (12.3)	218 (18.2)	<.001∗	428 (45.7)	294 (53.9)	.004∗
CFAE	30 (1.3)	21 (1.3)	.981	11 (0.8)	10 (0.8)	.825	19 (2.0)	11 (2.4)	.668
Mitral isthmus line (anterior)	81 (3.4)	100 (6.0)	<.001∗	24 (1.7)	46 (3.8)	<.001∗	57 (6.1)	54 (11.7)	<.001∗
Mitral isthmus line (lateral)	52 (2.2)	38 (2.3)	.813	17 (1.2)	26 (2.2)	.043∗	35 (3.7)	12 (2.6)	.267
Roof line	210 (8.8)	165 (9.9)	.217	72 (5.0)	67 (5.6)	.469	138 (14.7)	98 (21.2)	.002∗
Isolation/homogenization of fibrosis	43 (1.8)	40 (2.4)	.179	10 (0.7)	18 (1.5)	.042∗	33 (3.5)	22 (4.8)	.262
LAA focal	10 (0.4)	9 (0.5)	.572	3 (0.2)	5 (0.4)	.530	7 (0.7)	4 (0.9)	1
LAA isolation	12 (0.5)	14 (0.8)	.185	10 (0.7)	13 (1.0)	.279	2 (0.21)	1 (0.2)	.987
Crista	8 (0.3)	3 (0.2)	.535	5 (0.3)	3 (0.3)	.933	3 (0.3)	0 (0.0)	.555
Carina	173 (7.2)	120 (7.2)	.984	78 (5.4)	67 (5.6)	.808	95 (10.1)	53 (11.5)	.446
Fossa ovalis	6 (0.3)	5 (0.4)	1	5 (0.3)	4 (0.3)	1	1 (0.1)	1 (0.2)	.552
Eustachian ridge	2 (0.1)	4 (0.2)	.388	1 (0.1)	2 (0.2)	.868	1 (0.1)	2 (0.4)	.255
Ganglia ablation	20 (0.8)	16 (1.0)	.674	13 (0.9)	10 (0.8)	.866	7 (0.7)	6 (1.3)	.475
Vein of Marshall	65 (3.8)	36 (3.3)	.446	19 (2.1)	21 (2.8)	.316	46 (5.9)	15 (4.2)	.245
SVC	35 (1.4)	46 (2.7)	.004∗	20 (1.3)	36 (2.9)	.004∗	15 (1.6)	10 (2.1)	.445
CS	56 (2.3)	58 (3.4)	.034∗	23 (1.5)	40 (3.2)	.003∗	33 (3.4)	18 (3.8)	.710
AVNRT pathway	59 (2.4)	43 (2.5)	.825	44 (2.9)	40 (3.2)	.668	15 (1.6)	3 (0.6)	.140
RF parameters									
Index guided (any)	2115 (88.8)	1446 (88.5)	.799	1289 (90.3)	1037 (88.9)	.246	826 (86.4)	409 (87.4)	.604
Rate of procedures done under high-power short-duration ablation	2361 (95.4)	1626 (94.2)	.072	1415 (93.3)	1169 (93.0)	.725	946 (98.6)	457 (97.2)	.061
Anterior RF max power	47.83 ± 3.89	47.81 ± 3.81	.863	47.33 ± 4.27	47.55 ± 3.94	.162	48.6 ± 3.06	48.49 ± 3.37	.507
Anterior RF max force	19.49 ± 6.63	19.96 ± 13.9	.160	19.6 ± 7.07	19.65 ± 6.78	.857	19.34 ± 5.93	20.73 ± 23.48	.213
Anterior RF index	497.53 ± 46.24	496.53 ± 42.83	.526	495.86 ± 50.05	494.68 ± 46.82	.574	499.99 ± 39.85	500.87 ± 31.23	.695
Posterior RF max power	45.76 ± 5.81	45.76 ± 5.81	.285	45.21 ± 6.06	45.62 ± 5.67	.069	46.6 ± 5.3	46.82 ± 5.45	.472
Posterior RF max force	17.18 ± 7.23	18.3 ± 18.69	.023∗	17.56 ± 7.58	18.12 ± 17.91	.289	16.62 ± 6.65	18.74 ± 20.49	.032
Posterior RF index	393.19 ± 41.89	392.24 ± 40.19	.513	392.8 ± 44.75	392.11 ± 44.11	.721	393.76 ± 37.29	392.55 ± 29.02	.567
Max esophageal temperature	36.73 ± 5.8	36.99 ± 5.17	.200	36.79 ± 5.67	37.22 ± 4.6	.052	36.64 ± 6.03	36.28 ± 6.61	.408
Procedure times, min									
Total procedure time	92.00 (74.00–119.00)	90 (69.50–121.50)	.007∗	89.00 (74.00–115.5)	83.00 (67.00–117.5)	.059	96.50 (74.75–128.25)	101.00 (84.00–139.75)	.824
PVI ablation time	18.23 (14.43–23.00)	16.37 (13.10–20.33)	<.001∗	18.52 (13.99–23.58)	16.08 (12.49–19.96)	<.001∗	18.01 (14.73–22.49)	17.18 (14.49–21.40)	<.001∗
RF time	23.62 (17.75–30.43)	22.35 (16.47–30.13)	<.001∗	22.18 (17.02–30.10)	19.97 (15.72–27.96)	<.001∗	25.59 (18.48–30.59)	26.00 (19.96–34.47)	.890
Fluoroscopy	0 (0–0.7)	0 (0–0.5)	.200	0 (0–0.3)	0 (0–0.3)	.663	0 (0–1.1)	0 (0–1.1)	.948
Minimal Fluoroscopy	1953 (78.9)	1404 (81.5)	.043∗	1241 (81.9)	1051 (83.9)	.173	712 (74.2)	353 (75.1)	.725
Same-day discharge	1663 (79.0)	1016 (72.5)	<.001∗	921 (80.2)	706 (75.7)	.014∗	742 (77.6)	310 (66.1)	<.001∗
Procedure events									
Carina ablation	313 (12.6)	209 (12.1)	.599	154 (10.2)	131 (10.4)	.820	159 (16.6)	78 (16.6)	.994
AF terminated via DCCV	424 (17.1)	255 (14.8)	.040∗	119 (7.8)	85 (6.8)	.275	305 (31.8)	170 (36.2)	.100
Drug- or pacing-induced arrhythmias	666 (26.9)	526 (30.5)	.012∗	386 (25.5)	355 (28.2)	.100	280 (29.2)	171 (36.4)	.006∗

Values are presented as means ± standard deviations and numbers (percentages).

AF = atrial fibrillation; AFL = atrial flutter; AT = atrial tachycardia; AVNRT = atrioventricular nodal reentry tachycardia; CFAE = complex fractionated atrial electrogram; CS = coronary sinus; CT = computed tomography; CTI = cavotricuspid isthmus; DCCV = direct current cardioversion; FPI = first-pass isolation; GA = general anesthesia; HFLV = high-flow low-volume ventilation; ICE = intracardiac echocardiography; JET = jet ventilation; LA = left atrial; LAA = left atrial appendage; max = maximum; PAF = paroxysmal atrial fibrillation; PsAF = persistent atrial fibrillation; PVI = pulmonary vein isolation; RF = radiofrequency; SM = substrate modification; SVC = superior vena cava; SVT = supraventricular tachycardia; TEE = transesophageal echocardiography.

∗ Statistically significant.

† LA volume index: defined as the LA volume divided by body surface area (mL/m^2^).

Despite undergoing more complex ablation procedures, women had shorter total procedure times than men (90 [69.50–121.50] vs 92.00 [74.00–119.00] minutes; *P* = .007). Similarly, PV ablation time was shorter in women (16.37 [13.10–20.33] vs 18.23 [14.43–23.00] minutes; *P* < .001), as well as RF time (22.35 [16.47–30.13] vs 23.62 [17.75–30.43] minutes; *P* < .001). Nevertheless, these differences did not lead to clinically significant differences in acute efficacy and safety outcomes. Fluoroscopy usage also differed, with more women undergoing minimal fluoroscopy procedures (81.5% vs 78.9%; *P* = .043). However, fewer women were discharged on the same day than men (72.5% vs 79.0%; *P* < .001). There were significant differences in the use of high-frequency low-tidal-volume (HFLTV) ventilation and GA between sexes. Women treated for PAF were more likely to receive HFLTV ventilation (43.1% vs 38.3%; *P* = .016) and had a slightly lower rate of receiving GA (82.6% vs 85.4%; *P* = .049) than men (Figure 1, Table 2).

### Acute success

In this study, acute procedural success, measured by first-pass isolation (FPI) of both PVs, was compared between men and women using odds ratios (OR). After adjusting for confounders such as age and comorbidities, a generalized linear mixed model showed no significant relationship between sex and FPI. Nonetheless, women with PsAF were significantly less likely to achieve left-sided FPI (adjusted OR 0.644; 95% CI 0.424–0.978; *P* = .039) (Table 3). Women were less likely to require electrical cardioversion to terminate AF (14.8% vs 17.1%; *P* = .040), but experienced higher rates of drug- or pacing-induced arrhythmias, particularly in PsAF (36.4% in women vs 29.2% in men; *P* = .006). Other metrics, such as carina ablation and post-PVI waiting times, were similar across sexes (Table 2).

**Table 3:** Acute success assessment by AF type

Overall				PAF				PsAF				
FPI	Unadjusted OR (95% CI)	*P* value	Adjusted OR (95% CI)	*P* value	Unadjusted OR (95% CI)	*P* value	PAF adjusted OR (95% CI)	*P* value	Unadjusted OR (95% CI)	*P* value	Adjusted OR (95% CI)	*P* value
Both∗	0.844 (0.737–0.966)	.014	0.868 (0.725–0.039)	.121	0.848 (0.719–0.999)	.049	0.851 (0.683–1.059)	.148	0.850 (0.666–1.086)	.193	0.950 (0.671–0.347)	.542
Right∗	0.925 (0.798–1.073)	.306	0.976 (0.804–0.186)	.804	0.869 (0.719–1.050)	.150	0.896 (0.705–1.139)	.378	0.942 (0.743–1.194)	.612	0.918 (0.590–0.427)	.771
Left∗	0.807 (0.676–0.963)	.018	0.891 (0.734–0.014)	.091	0.864 (0.695–1.074)	.193	0.924 (0.705–1.236)	.608	0.746 (0.546–1.022)	.068	0.663 (0.424–0.041)	.039

PAF				PsAF		
Men		Women	*P* value	Men	Women	*P* value
Bilateral FPI	1573 (66.3)	1154 (69.9)	.015	970 (66.6)	850 (70.2)	.049
Right FPI	1806 (75.7)	1278 (77.1)	.302	1113 (76.0)	938 (77.1)	.503
Left FPI	1995 (83.5)	1434 (86.3)	.017	1243 (84.7)	1053 (86.5)	.188

Values are presented as numbers (percentages). Adjusted for age, body mass index, vascular disease, heart failure, hypertension, transient ischemic attack/cerebrovascular accident, diabetes, and sleep apnea.

AF = atrial fibrillation; CI = confidence interval; FPI = first-pass isolation; OR = odds ratio; PAF = paroxysmal atrial fibrillation; PsAF = persistent atrial fibrillation.

∗ Men as reference.

### Complications

In the overall population, procedural complication rates were comparable between sexes (2.0% [women] vs 1.6% [men]; *P* = .274). However, the most significant difference was observed in vascular access complications, with women more frequently affected than men (0.6% vs 0.2%; *P* = .024). No notable sex differences were found in overall complication rates in the PAF cohort. In contrast, women with PsAF experienced higher overall complication rates (3.8% vs 1.5%; *P* = .004), primarily driven by vascular access complications (1.3% vs 0.1%; *P* = .010). Other major complications, including cardiac tamponade, stroke, and phrenic nerve injury, were similarly distributed between sexes (Table 4).

**Table 4:** Procedural complications by sex and AF type

Complications	Overall			PAF			PsAF		
	Men	Women	*P* value	Men	Women	*P* value	Men	Women	*P* value
Procedural complications (any)	39 (1.6)	35 (2.0)	.274	25 (1.6)	17 (1.4)	.524	14 (1.5)	18 (3.8)	.004∗
Cardiac tamponade/pericardial effusion	7 (0.3)	7 (0.4)	.498	3 (0.2)	5 (0.4)	.534	4 (0.4)	2 (0.4)	1
TIA	0 (0.0)	1 (0.1)	.411	0	0	-	0 (0.0)	1 (0.2)	.329
Stroke	1 (0.0)	2 (0.1)	.745	1 (0.1)	1 (0.1)	1	0 (0.0)	1 (0.2)	.329
Atrioesophageal fistula	0	0	-	0	0	-	0	0	
Death	2 (0.1)	0 (0.0)	.516	1 (0.1)	0 (0.0)	1	1 (0.1)	0 (0.0)	1
Esophageal injury	2 (0.1)	0 (0.0)	.516	2 (0.1)	0 (0.0)	.504	0	0	
Vagal nerve injury	0	0	-	0	0		0	0	
Phrenic nerve injury	0 (0.0)	2 (0.1)	.169	0 (0.0)	1 (0.1)	.453	0 (0.0)	1 (0.1)	.329
Pericarditis									
Pulmonary vein stenosis	3 (0.1)	0 (0.0)	.390	3 (0.1)	0 (0.0)	.318	0	0	
Vascular access (any)	5 (0.2)	11 (0.6)	.024∗	4 (0.3)	5 (0.4)	.778	1 (0.1)	6 (1.3)	.010∗
Hematoma	3 (0.1)	3 (0.2)	.977	2 (0.1)	2 (0.2)	1	1 (0.1)	1 (0.2)	.550
AV fistula	0 (0.0)	1 (0.1)	.411	0	0	-	0 (0.0)	1 (0.2)	.329
Pseudoaneurysm	1 (0.0)	7 (0.4)	.021	1 (0.1)	3 (0.2)	.490	0 (0.0)	4 (0.9)	.020∗
Bleeding†	3 (0.1)	3 (0.2)	.977	1 (0.1)	0 (0.0)	1	2 (0.2)	3 (0.6)	.415

Values are presented as numbers (percentages).

AF = atrial fibrillation; AV = arteriovenous; PAF = paroxysmal atrial fibrillation; PsAF = persistent atrial fibrillation; TIA = transient ischemic attack.

∗ Statistically significant.

† Bleeding was defined as bleeding occurring within the first 72 hours after the procedure that requires treatment with transfusion or results in a ≥20% decrease in hematocrit or a ≥2 g decrease in hemoglobin.

### Clinical outcomes after AF ablation

At the 3-month follow-up, both men and women showed similar arrhythmia recurrence rates (12.5% vs 11.4%; *P* = .296); however, women had a higher need for repeat ablations after the blanking period (7.2% vs 5.5%; *P* = .022), particularly in the PAF cohort (7.2% vs 4.8%; *P* = .009). By 12 months, significant sex-based differences emerged, with women exhibiting a higher overall arrhythmia recurrence rate (22.5% vs 18.6%; *P* = .002) and a greater proportion of symptomatic recurrences (11.7% vs 8.7%; *P* = .002). In particular, women exhibited a higher incidence of AF recurrence (16.6% vs 14.3% in men; *P* = .037), with significant differences within the PAF subgroup (15.1% in women vs 12.4% in men; *P* = .038). Atypical AFL showed a trend toward higher prevalence in women overall (3.1% in women vs 2.1% in men; *P* = .059).

Hospitalizations were also more frequent among women (4.8% vs 2.9%; *P* = .001), especially within the PAF subgroup (4.6% vs 2.6%; *P* = .004). Despite these higher recurrence and hospitalization rates, both sexes demonstrated significant improvements in quality of life, with more than 90% indicating better well-being at 12 months (*P* = .203). Furthermore, there was no significant difference in the discontinuation of AADs after the blanking period between men and women (93.6% vs 93.1%; *P* = .491). However, women were less likely to discontinue OACs compared with men (23.2% vs 31.8%; *P* < .001), particularly in the PsAF cohort (13.1% vs 22.1%; *P* < .001) (Table 5).

**Table 5:** 3- and 12-month clinical outcomes by sex and AF type

Clinical outcomes	Overall			PAF			PsAF		
	Men	Women	*P* value	Men	Women	*P* value	Men	Women	*P* value
3-mo outcome									
Arrhythmia recurrence	292 (12.5)	187 (11.4)	.296	116 (8.2)	101 (8.5)	.746	176 (19.4)	86 (19.2)	.935
Off AAD	1642 (70.7)	1187 (72.8)	.163	1062 (75.1)	889 (75.1)	.956	580 (64)	298 (66.5)	.365
Repeat ablation after the blanking period	135 (5.5)	124 (7.2)	.022∗	73 (4.8)	90 (7.2)	.009∗	62 (6.5)	34 (7.2)	.585
12-mo outcome									
Arrhythmia recurrence assessments									
All arrhythmia recurrence	461 (18.6)	388 (22.5)	.002∗	244 (16.1)	251 (20.0)	.008∗	217 (22.6)	137 (29.1)	.007∗
Symptomatic recurrences	216 (8.7)	202 (11.7)	.002∗	111 (7.3)	127 (10.1)	.009∗	105 (10.9)	75 (16.0)	.007∗
Asymptomatic recurrences	123 (5.0)	85 (4.9)	.944	56 (3.7)	47 (3.7)	.950	67 (6.97)	38 (8.1)	.455
Type of recurrent arrhythmia									
AF	353 (14.3)	287 (16.6)	.037∗	188 (12.4)	190 (15.1)	.038∗	165 (17.2)	97 (20.6)	.47
Typical flutter	31 (1.3)	18 (1.0)	.532	13 (0.9)	9 (0.7)	.676	18 (1.8)	9 (1.9)	.961
Atypical flutter	53 (2.1)	53 (3.1)	.059	24 (1.6)	31 (2.5)	.097	29 (3.0)	22 (4.7)	.113
AT	19 (0.8)	26 (1.5)	.022∗	16 (1.1)	18 (1.4)	.370	3 (0.3)	8 (1.7)	.005∗
Cardioversions since ablation	115 (4.6)	83 (4.8)	.810	31 (2.0)	38 (3.0)	.100	84 (8.7)	45 (9.6)	.613
Hospitalizations	72 (2.9)	83 (4.8)	.001∗	39 (2.6)	58 (4.6)	.004∗	33 (3.4)	25 (5.3)	.091
Off AAD	2304 (93.1)	1617 (93.6)	.491	1428 (94.2)	1185 (94.3)	.931	876 (91.3)	432 (91.9)	.716
Off OACs	757 (31.8)	383 (23.2)	<.001∗	552 (37.9)	324 (26.9)	<.001∗	205 (22.1)	59 (13.1)	<.001∗
Self-reported QoL/symptoms (feeling better)	1754 (92.3)	1241 (93.4)	.203	1122 (93.5)	914 (93.8)	.747	632 (90.2)	327 (92.4)	.237
Clinical success†	2074 (88.4)	1368 (84.1)	<.001∗	1283 (88.8)	1005 (84.8)	.003∗	791 (87.7)	363 (82.3)	.008∗

Values are presented as numbers (percentages).

AAD = antiarrhythmic drug; AF = atrial fibrillation; AT = atrial tachycardia; OAC = oral anticoagulant; PAF = paroxysmal atrial fibrillation; PsAF = persistent atrial fibrillation; QoL = quality of life.

∗ Statistically significant.

† Clinical success is defined as follows: (1) for PAF, absence of atrial arrhythmias with either no AAD, reduced AAD dose, or effectiveness of a previously ineffective dose; (2) for PsAF, absence of atrial arrhythmias or asymptomatic, self-terminating arrhythmia recurrence, with either no AAD, reduced AAD dose, or effectiveness of a previously ineffective dose.

### Arrhythmia recurrence and impact of ablation strategy

**Figure 2:**
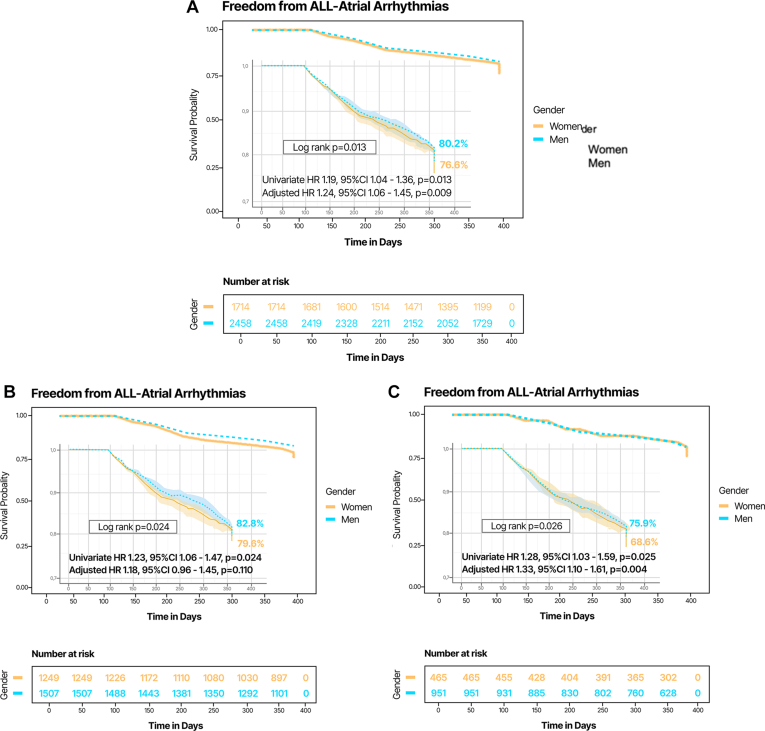
Arrhythmia-free survival in women and men for the entire patient cohort (**A**), and separated by AF subtype: paroxysmal AF (**B**) vs persistent AF (**C**). *∗*Adjusted HR for age, body mass index, AF type, comorbidities, left atrial volume index, and physicians. AF = atrial fibrillation; CI = confidence interval; HR = hazard ratio.

Kaplan-Meier analysis revealed that women had significantly higher rates of arrhythmia recurrence than men at the 12-month follow-up (log-rank test, *P* = .013) (Figure 2). This finding was further supported by Cox proportional hazards models, which indicated a higher recurrence risk for women across all univariate models. After adjusting for covariates, women had a significantly higher risk of arrhythmia recurrence across the overall cohort, with an adjusted HR (aHR) of 1.24; 95% CI 1.06–1.45; *P* = .009. In the PAF cohort, women showed a trend toward higher recurrence rates, but this difference did not reach statistical significance (HR 1.18; 95% CI 0.96–1.45; *P* = .110). However, in the PsAF cohort, women demonstrated a significantly elevated recurrence risk (HR 1.33; 95% CI 1.10–1.61; *P* = .004) (Figure 2, Supplemental Table 1).

**Figure 3:**
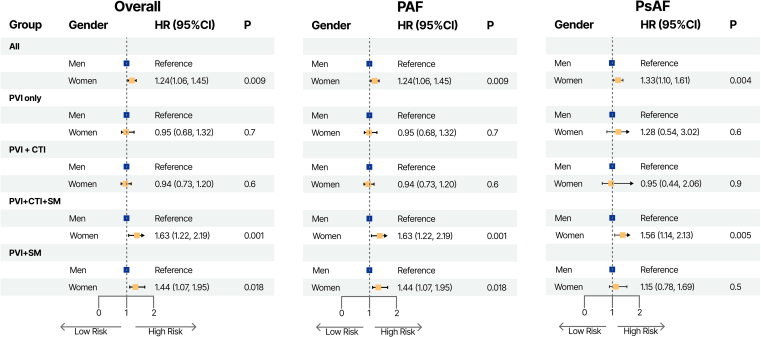
Risk of arrhythmia recurrence with different ablation strategies during long-term follow-up in the entire cohort, PAF cohort, and PsAF cohort. ∗Adjusted HR for age, body mass index, AF type, comorbidities, left atrial volume index, and physicians. AF = atrial fibrillation; CI = confidence interval; CTI = cavotricuspid isthmus ablation; HR = hazard ratio; PAF = paroxysmal atrial fibrillation; PVI = pulmonary vein isolation; PsAF = persistent atrial fibrillation; SM = substrate modification.

Subgroup analysis based on ablation strategy indicated that women undergoing procedures involving SM had a higher risk of recurrence than men. In the PVI + SM group, the fully aHR for women was 1.44; 95% CI 1.07–1.95; *P* = .018, and in the PVI + CTI + SM group, this risk was even higher (aHR 1.63; 95% CI 1.22–2.19; *P* < .001). Conversely, no significant sex differences were found in patients who underwent PVI only (aHR 0.92; 95% CI 0.71–1.19; *P* = .5) or PVI + CTI (aHR 0.97; 95% CI 0.73–1.30; *P* = .9) (Figure 3, Supplemental Table 1).

### Redo procedures

Data on repeat ablation procedures were available for 242 patients, including 132 men and 110 women. 7 patients underwent a third ablation procedure. At the time of the first repeat procedure, women were more likely than men to present with all PVs remaining isolated (54.5% vs 40.9%; *P* = .034) and tended to present in sinus rhythm more frequently (60.0% vs 47.8%; *P* = .057). Right-sided PV reconnection was more commonly observed in men than in women during redo procedures. In particular, reconnection of the right superior PV occurred more frequently in men (42.4% vs 28.2%; *P* = .022), as did reconnection of the right inferior PV (37.9% vs 24.5%; *P* = .027). There were no significant differences in SM strategies performed during repeat procedures between men and women (*P* = .447). Particularly, PWI was performed in 62.9% of men and 56.1% of women (*P* = .306).

## Discussion

This study from the REAL-AF registry highlights key sex-related differences in ablation outcomes for PAF and PsAF. Women were older, had higher CHA_2_DS_2_-VASc scores, and underwent more complex ablations beyond PVI, including additional SM. Despite these differences, women achieved similar or slightly higher rates of FPI with shorter procedure times than men. However, long-term follow-up showed higher rates of atrial arrhythmia recurrence, symptomatic episodes, and hospitalizations after ablation, particularly in PsAF, with female sex being an independent predictor of recurrence. Interestingly, women undergoing SM had significantly higher risks of recurrence than men across both PAF and PsAF populations. Although overall complication rates were similar, women had more vascular access complications. These findings suggest the need for tailored ablation strategies in women to address their higher recurrence risk.

### Sex-specific patient characteristics

Under-referral of women for CA and their referral at later stages of AF progression have been highlighted.^3^^,^^15^^,^^21^^,^^22^ In our cohort, fewer women underwent CA, even though consecutive patient recruitment was encouraged. This trend aligns with national data, where female representation in CA remains <30%, regardless of region or center experience.^13^ Although this could partly reflect the higher AF burden in men,^23^^,^^24^ the fact that women in our study were older at the time of ablation raises concerns about potential biases in treatment referral. However, the comparable diagnosis-to-ablation interval between sexes in our cohort suggests that the older age of women at the time of RFCA may not be caused by delayed referral but rather a reflection of the natural history of AF, where women typically develop the condition later in life.^21^^,^^25^^,^^26^ This finding is consistent with epidemiologic data showing that women tend to develop AF approximately a decade later than men.^27^ In addition, women in this cohort exhibited higher CHA_2_DS_2_-VASc scores owing to factors such as older age and stroke or transient ischemic attack history. The higher baseline CHA_2_DS_2_-VASc scores in women could partly explain their lower rates of OAC discontinuation, given that clinicians may be more cautious in stopping OAC therapy in patients with a higher stroke risk and more frequent symptomatic recurrences. Paradoxically, they had significantly lower rates of cardiovascular comorbidities such as hypertension, vascular disease, and diabetes than men. This complex pattern highlights the interplay of age, sex, and stroke risk in AF management.

### Higher long-term recurrence in women: Potential mechanisms

Previous studies have yielded inconsistent findings regarding sex differences in long-term procedural success after AF ablation.^13^^,^^15^^,^^22^^,^^25^ Although several reports suggest that women experience higher recurrence rates after ablation, larger studies in patients have not reported a difference in ablation outcomes.^22^^,^^28^^,^^29^ Our study, one of the largest AF ablation cohorts, found that women were significantly more likely to experience recurrence. This occurred despite several favorable prognostic factors in women, such as fewer cardiovascular comorbidities, higher PAF representation, and better left ventricular ejection fraction with smaller LA dimensions. Kaplan-Meier analysis showed that although acute success and 3-month outcomes were similar, long-term recurrence rates in women consistently exceeded those in men. Female sex remained an independent predictor of recurrence, especially in patients with PsAF. These findings suggest that sex-specific structural and electrophysiological differences, such as increased atrial fibrosis or scarring, may underlie the elevated recurrence risk in women.

### Atrial remodeling and fibrosis

Our findings suggest more aggressive ablation strategies in women, moving beyond earlier empirical approaches as previously suggested.^4^^,^^30^ Advanced mapping techniques revealed a significantly higher prevalence of LA scarring, abnormal electroanatomic activity, and more frequent atypical AFL in women. These observations indicate that the need for additional SM and linear ablation stems from more advanced atrial remodeling, rather than clinical discretion. Men, conversely, were more likely to undergo CTI ablation owing to the higher prevalence of typical AFL. The sex-specific procedural strategies likely reflect anatomic and electrophysiological distinctions, with women more often requiring interventions beyond PVI owing to atypical arrhythmias and more complex atrial remodeling.

One possible explanation for these findings is that women, compared with men, are more likely to (1) convert from AF to roof- or mitral-dependent AFL during PVI or (2) have atypical AFL induced either before or after PVI. In such cases, the use of adjunctive ablation lines, such as roof or mitral isthmus lines, is supported by current recommendations.^31^^,^^32^ Although sex differences in atypical AFL inducibility and maintenance have not been extensively characterized, previous studies have shown that women exhibit different atrial characteristics compared with men.^33^^,^^34^ Moreover, women are known to have a higher prevalence of atrial fibrosis, which alters the atrial substrate and fosters conditions favorable for reentrant circuits, increasing the likelihood of atypical AFL.^35^ The presence of fibrosis and scar tissue often compromises the effectiveness of standard PVI and may also diminish the success of SM strategies. For instance, adequate reduction of contiguous atrial mass is essential for long-term success in AF ablation.^36^ The higher recurrence rates observed in women after SM suggest that current approaches may not be achieving sufficient mass reduction, particularly in areas of extensive fibrosis. This highlights the need for more tailored ablation strategies that not only address arrhythmia triggers but also emphasize debulking of electrically active tissue, especially in patients with complex atrial substrates.

### Non-PV triggers and SM

The higher prevalence of non-PV triggers in women^37^ may further explain their increased recurrence risk. Non-PV triggers, typically arising from discrete anatomic structures such as mitral and tricuspid periannular regions, crista terminalis, LA PW, LA appendage, and various thoracic veins (eg, superior vena cava, coronary sinus, and ligament of Marshall),^38^ have been shown to contribute to arrhythmia recurrence.^39^^,^^40^ Notably, our results demonstrated that women more frequently required ablations targeting these areas, reinforcing the role of non-PV triggers in female patients. In addition, women had higher rates of drug- or pacing-induced arrhythmias after PVI, emphasizing the complexity of their arrhythmogenic substrates.

Several mechanisms may contribute to the observed sex differences. Women with AF have been reported to demonstrate greater atrial fibrosis and structural remodeling, which may translate into a more complex arrhythmogenic substrate requiring additional ablation strategies. Hormonal influences, differences in atrial size, and potential delays in referral for rhythm control therapies may further contribute to these findings. These findings highlight the potential role of precision medicine approaches, including advanced atrial imaging for fibrosis characterization and earlier detection protocols, to better tailor ablation strategies and improve outcomes in women.

Furthermore, although early work has provided foundational insights into sex differences in AF ablation outcomes,^21^ the REAL-AF registry advances the field through a prospective design, greater female representation, and detailed analyses of ablation techniques such as high-power short-duration, zero-fluoroscopy workflows and sex-specific recurrence risks stratified by AF type. These results offer contemporary, real-world insights that are generalizable to current clinical practice.

### Study limitations

This study has several limitations. First, as an observational registry, the analysis is inherently subject to selection bias and the potential influence of unmeasured confounders. Although the study accounted for physician variability in multivariable models and adjusted for baseline differences between sexes, residual confounding may remain. Patient selection and ablation approaches were at the discretion of participating centers, which may have biased the results, and the registry did not capture patients who were not referred for or declined ablation. Second, rhythm surveillance varied across centers, with limited continuous monitoring and incomplete compliance with 6- and 12-month assessments. This may have contributed to the underdetection of asymptomatic recurrences, particularly in patients without cardiac implantable devices. In addition, longer-term follow-up beyond 1 year could yield different results. Third, the analysis was limited to patients treated with RF energy. Given that pulsed-field ablation uses a different mechanism and procedural considerations, these findings may not be generalized to nonthermal ablation approaches. Finally, data on the type of vascular closure technique were not collected; we acknowledge that this factor could influence discharge times and the risk of groin complications. Standardized assessments of quality of life were not performed, limiting the evaluation of patients’ physical and mental well-being throughout the study period. Furthermore, AF burden could not be systematically assessed owing to variability in rhythm monitoring techniques across centers, including limited continuous monitoring and differing follow-up protocols.

## Conclusion

Our findings demonstrated significant sex-based differences in AF ablation. Women present at an older age with more comorbidities and undergo more extensive lesion sets, yet still experience higher long-term recurrence and hospitalization rates. This suggests a more diffuse arrhythmogenic substrate in women, particularly those with PsAF, with greater non-PV triggers and advanced atrial remodeling that may not be fully addressed by current strategies. These disparities highlight the need for tailored ablation approaches in women. Future studies should focus on characterizing atrial substrate, non-PV trigger burden, and optimal lesion set design to improve long-term outcomes.

## Disclosures

Drs Jose Osorio and Paul C. Zei have received consulting and research support from Johnson & Johnson MedTech Electrophysiology. Dr Sandeep K. Goyal is a consultant for Johnson & Johnson MedTech Electrophysiology and Medtronic. Dr Benjamin D’Souza is a consultant for Johnson & Johnson MedTech Electrophysiology. Dr Anil Rajendra received consulting fees from Johnson & Johnson MedTech Electrophysiology and Boston Scientific. Dr Joshua Silverstein reports consulting for Johnson & Johnson MedTech Electrophysiology. All other authors have no conflicts of interest to disclose.

## Footnotes

Appendix Supplementary data associated with this article can be found in the online version at https://doi.org/10.1016/j.hroo.2026.04.005.
