# Supplementary material for: Real-world data of sex differences in atrial fibrillation catheter ablation: Insights from a prospective multicenter registry
Source: Heart Rhythm O2. 2026 Apr 13;7(7):1259–73. doi: 10.1016/j.hroo.2026.04.005 (PMC13390002; doi:10.1016/j.hroo.2026.04.005)
Supplement: Supplementary Appendix [file mmc1.pdf]

Supplemental Appendix

Supplemental **Figure S1.** Cohort selection diagram……………………………………………………………….….**Page 2**

Supplemental **Figure S2.** Ablation strategy and procedure time by sex during ablation for paroxysmal atrial fibrillation cohort….………..…………………………………………………..……………………………………**Page 3**

Supplemental **Figure S3.** Ablation strategy and procedure time by sex during ablation for persistent atrial fibrillation cohort.……………………………………………………...…………………………………….……….**Page 4**

Supplemental **Table 1.** Example Reasons for OAC Discontinuation…………………………..…………………..…..…..**Page 5**

Supplemental **Table 2.** Long-term outcome by Sex and AF type……………………………..…………………..……….**Page 6**

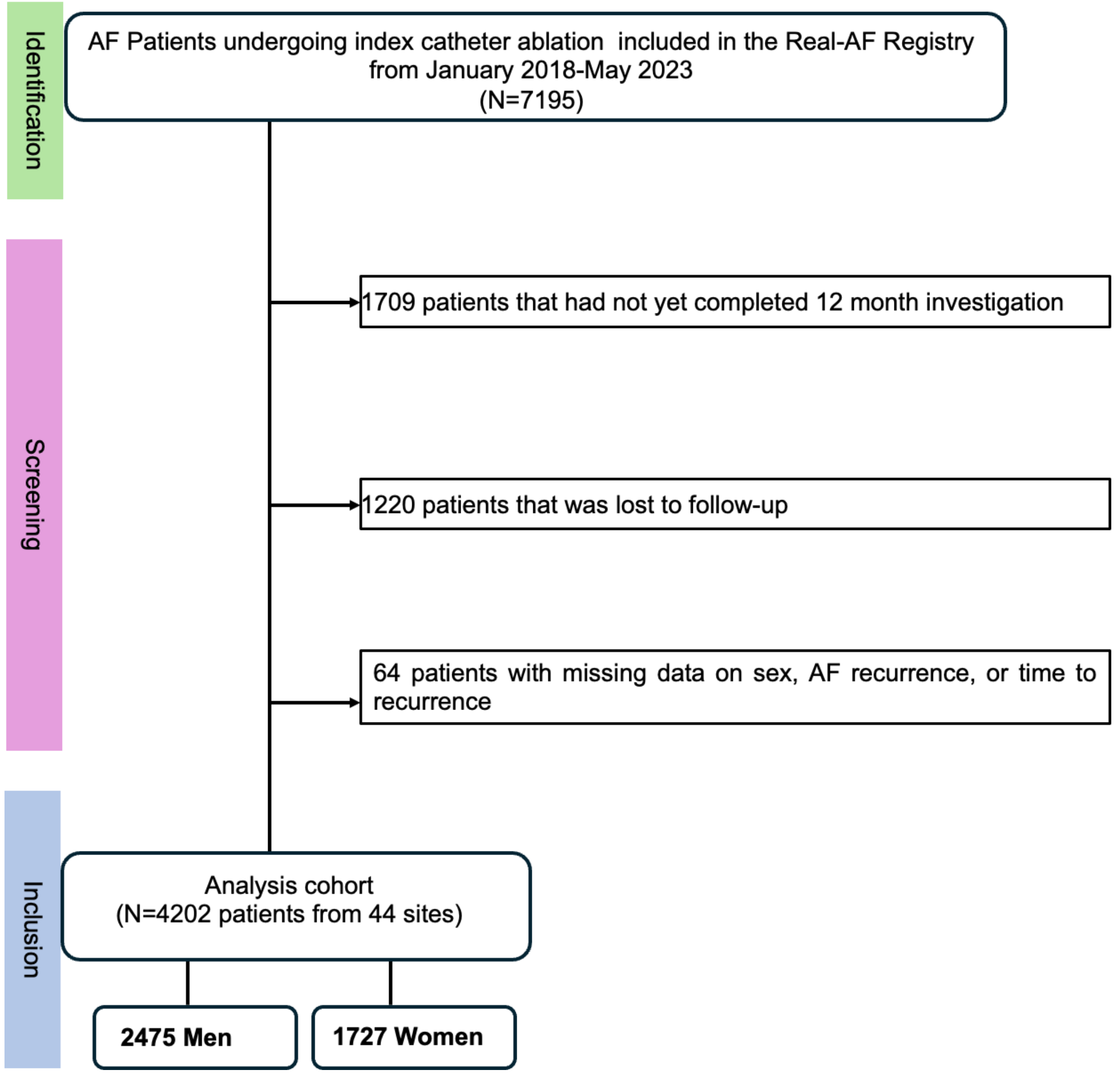

Figure S1. Cohort selection diagram.

Inclusion and exclusion criteria used to select analysis cohort. AF indicates atrial fibrillation including both paroxysmal and persistent AF.

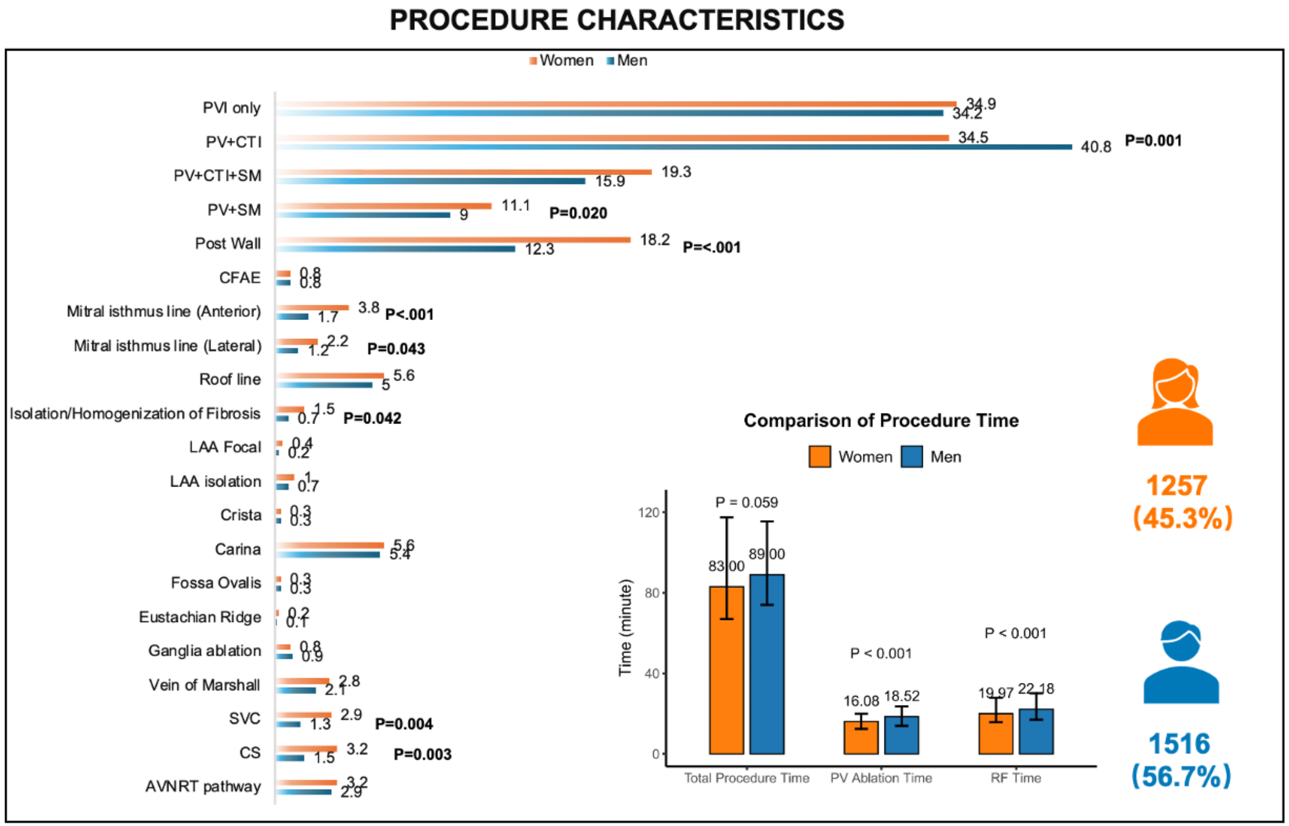

Figure S2. Ablation strategy and procedure time by sex during ablation for paroxysmal atrial fibrillation cohort.

Proportion of patients receiving each strategy and additional lesions beyond pulmonary vein isolation (PVI) in the REAL-AF registry. CTI, cavotricuspid isthmus; SM, substrate modification; CFAE, complex fractionated atrial electrograms; LAA, left atrial appendage; SVC, superior vena cava; CS, coronary sinus; PV, pulmonary vein.

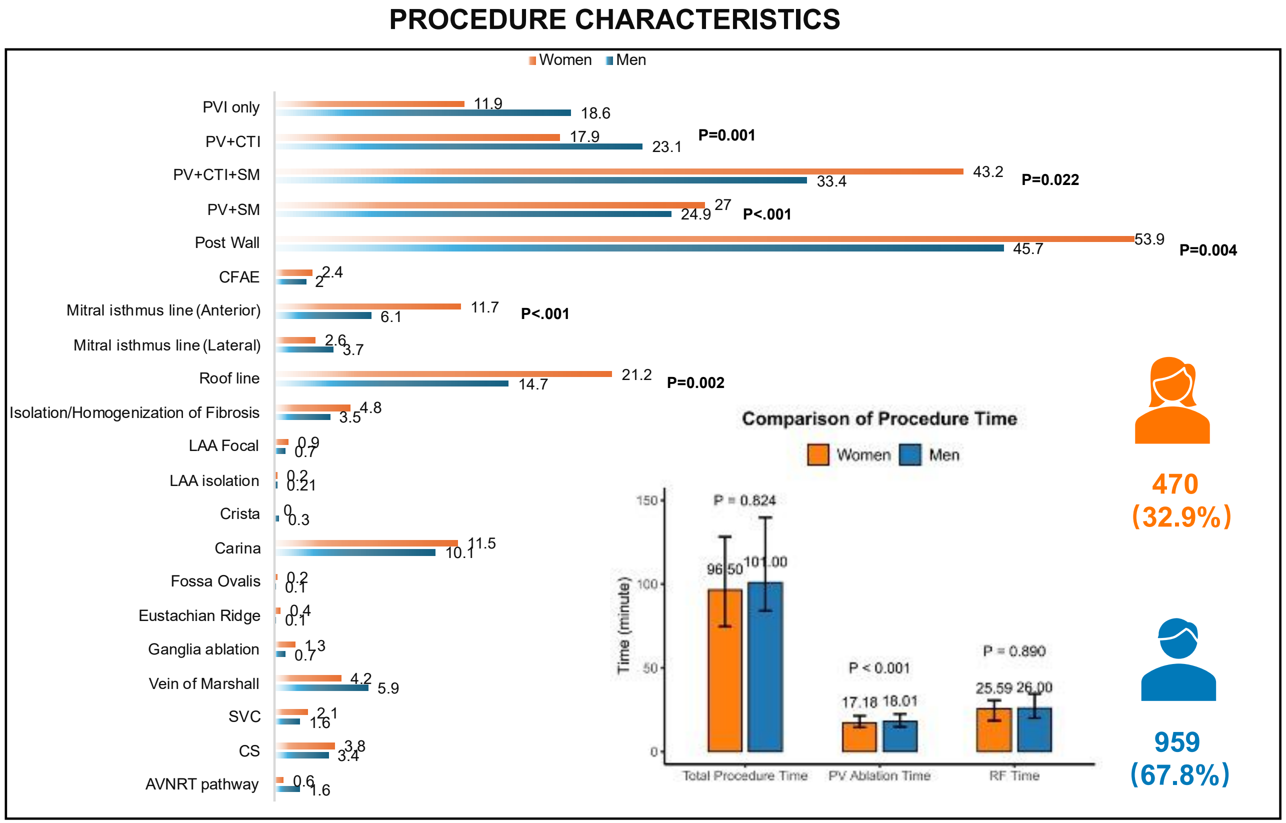

Figure S3. Ablation strategy and procedure time by sex during ablation for persistent atrial fibrillation cohort.

Proportion of patients receiving each strategy and additional lesions beyond pulmonary vein isolation (PVI) in the REAL-AF registry. CTI, cavotricuspid isthmus; SM, substrate modification; CFAE, complex fractionated atrial electrograms; LAA, left atrial appendage; SVC, superior vena cava; CS, coronary sinus; PV, pulmonary vein.

Supplemental Table 1. Example Reasons for OAC Discontinuation

**Example Reasons for OAC Discontinuation**
CHADS-VASc = 0; stopped at 90 days
GI bleed; Watchman placed
nosebleeds. received watchman device
Patient preference; on aspirin
Per PI discretion
Intracranial hemorrhage history
Low stroke risk; C2V = 1
Self-discontinued
Not on anticoagulation due to cost
High fall risk
No new complaints no chest pain no syncope
not on anticoagulation d/t liver cirrhosis

Supplemental Table 2. Long-term outcome by Sex and AF type

	Overall			PAF			PsAF		
	Model1	Model2	Model3	Model1	Model2	Model3	Model1	Model2	Model3
	HR 95%CI, p	HR 95%CI, p	HR 95%CI, p	HR 95%CI, p	HR 95%CI, p	HR 95%CI, p	HR 95%CI, p	HR 95%CI, p	HR 95%CI, p
All	1.19(1.04, 1.36), 0.013	1.19(1.04, 1.37), 0.013	1.24(1.06, 1.45), 0.009	1.23(1.03, 1.47), 0.024	1.23(1.03, 1.46), 0.020	1.18(0.96, 1.45), 0.110	1.28(1.03, 1.59), 0.025	1.28(1.07, 1.54), 0.007	1.33(1.10, 1.61), 0.004
PVI only	0.92(0.71, 1.19), 0.5	0.92(0.70, 1.21), 0.5	0.95(0.68, 1.32), 0.7	0.92(0.68, 1.24), 0.6	0.92(0.70, 1.22), 0.6	0.85(0.62, 1.18), 0.3	1.25(0.72, 2.19), 0.4	1.25(0.66, 2.37), 0.5	1.28(0.54, 3.02), 0.6
PVI+CTI	0.97(0.73, 1.30), 0.9	0.97(0.78, 1.22), 0.8	0.94(0.73, 1.20), 0.6	1.02(0.73, 1.42), >0.9	1.02(0.75, 1.38), >0.9	0.91(0.66, 1.27), 0.6	0.92(0.50, 1.70), 0.8	0.92(0.48, 1.75), 0.8	0.95(0.44, 2.06), 0.9
PVI+CTI+SM	1.52(1.17, 1.96), 0.001	1.52(1.21, 1.90), <0.001	1.63(1.22, 2.19), <0.001	1.73(1.16, 2.59), 0.007	1.73(1.23, 2.43), 0.001	1.66(1.10, 2.49), 0.015	1.45(1.03, 2.05), 0.032	1.45(1.11, 1.91), 0.007	1.56(1.14, 2.13), 0.005
PVI+SM	1.41(1.03, 1.92), 0.030	1.41(1.05, 1.89), 0.023	1.44(1.07, 1.95), 0.018	2.06(1.21, 3.53), 0.008	2.06(1.20, 3.54), 0.009	1.99(1.06, 3.76), 0.033	1.23(0.82, 1.83), 0.3	1.23(0.84, 1.78), 0.3	1.15(0.78, 1.69), 0.5

*Reference group is men. Univariate and multivariate Cox proportional hazards model to evaluate risk of arrhythmia recurrence with different ablation strategies in the overall cohort, paroxysmal AF(PAF) cohort, and persistent AF(PsAF) cohort. Model 1: Univariate analysis; Model 2: Adjusted for physician; Model 3: Adjusted for Age, BMI, Type of AF (In All Patients), Vascular Disease, Heart Failure, Hypertension, TIA/CVA, Diabetes, AAD, LA Volume Index: LA/BSA and physicians.

Abbreviations: PVI, Pulmonary Vein Isolation; CTI, Cavotricuspid Isthmus; SM, Substrate Modification; HR, Hazard Ratio; CI, Confidence Interval.
